# Fatal and nonfatal firearm injuries in the eastern Democratic Republic of Congo: a hospital-based retrospective descriptive cohort study assessing correlates of adult mortality

**DOI:** 10.1186/s12873-021-00506-3

**Published:** 2021-10-12

**Authors:** Paul Munguakonkwa Budema, Roméo Bujiriri Murhega, Tshibambe Nathanael Tshimbombu, Georges Kuyigwa Toha, Fabrice Gulimwentuga Cikomola, Paterne Safari Mudekereza, Léon-Emmanuel Mubenga, Ghislain Maheshe-Balemba, Darck Cubaka Badesire, Ulrick Sidney Kanmounye

**Affiliations:** 1 Department of Surgery, Provincial General Reference Hospital of Bukavu, Bukavu, Democratic Republic of Congo; 2 Faculty of Medicine, Université Catholique de Bukavu, Bukavu, Democratic Republic of Congo; 3 Research Department, Association of Future African Neurosurgeons, 37B Avenue des Marais, Forgeron, Funa, Kinshasa, Democratic Republic of Congo; 4 Geisel School of Medicine at Dartmouth, 1 Rope Ferry Rd, Hanover, NH 03755 USA; 5 Department of Radiology, Provincial General Reference Hospital of Bukavu, Bukavu, Democratic Republic of Congo

**Keywords:** Barriers to care, Conflict, Democratic Republic of Congo, Firearm injury, Survival

## Abstract

**Introduction:**

The Eastern Democratic Republic of Congo (DRC) has been the battleground for multiple armed conflicts, resulting in many fatal and nonfatal firearm injuries (F&NFFIs). Chronic insecurity has stressed the health system’s resources and created barriers to seeking, reaching, and receiving timely care further increasing the F&NFFI burden. Our institution is the largest trauma center in the region and receives the bulk of F&NFFI cases. We aimed to identify correlates of mortality in Congolese F&NFFI patients.

**Methods:**

We included all F&NFFI patients admitted to our institution between 2017 and 2020. We extracted data from patient charts and admission logs. We identified mortality correlates using the two-sample t-test, Chi-square test, and multivariable regression analysis. A *P*-value of less than 0.05 was considered statistically significant.

**Results:**

This study included 814 adult patients, mostly male (86%) with an average age of 34.5 years and living 154.4 km away from the hospital on average. The most affected anatomical sites were the lower limbs (48.2%) and upper limbs (23.2%). The median length of stay was 34.0 days, and the in-hospital mortality rate was 3.6%. In addition, mortality was negatively correlated with diastolic blood pressure (*P* = 0.01), SaO_2_ (*P* < 0.001), and hemoglobin concentration (*P* = 0.002).

**Conclusion:**

F&NFFIs cause an enormous burden in the region, and mortality is correlated with some clinical and biological variables. Thus, the study findings will inform F&NFFI referral, triage, and management in low-resource and mass casualty settings.

## Background

Injury is one of the leading causes of morbidity and mortality worldwide [1]. Violence acts, especially during war and conflict, are among the top ten greatest contributors of injury globally [2]. There are more than 60 ongoing conflicts globally, and most are in low- and middle-income countries (LMICs) [3]. Six of these conflicts (10.0%) are in the Democratic Republic of Congo, and four of the six are based in the Eastern Democratic Republic of Congo. Four provinces make up the Eastern Democratic Republic of Congo: Ituri, North Kivu, South Kivu, and Tanganyika. The two Kivu regions are home to the Kivu conflict. This complex sociopolitical context can be traced back to the colonial era with the tense relations between the indigenous Kivu population and neighboring Rwandans [4]. The clashes escalated in 1994 following the mass migration of Rwandan refugees fleeing the genocide [4]. Over the years, the pockets of internal conflict birthed armed groups that splintered and proliferated. The lure of financial gain catalyzed the proliferation of armed groups - the armed groups earn more than USD 183 million annually from the illegal trade of tin, tantalum, tungsten, and gold [5]. The recurrent clashes have led to over 1.4 million internal displacements and nearly 12,000 deaths [4].

The primary cause of injury and death in the Kivu conflict is firearms, followed by stabbing/cutting weapons, blunt objects, and hand grenades [4]. Firearm injuries create permanent and temporary cavities as they disrupt the surrounding tissues [6]. Tissue disruption is directly proportional to the projectile’s energy, and most firearm injuries affect the extremities [6–8]. Firearm injuries to the extremities affect the soft tissue and bone primarily [9]. Firearm injuries cause burns, crush injury, lacerations, nerve injury, vascular injury, and volumetric muscle loss [10]. Also, these primary injuries can be complicated by secondary injuries - the most common of these being infection [10]. Secondary injuries can be prevented or managed if the patient receives appropriate care on-site, prompt transfer to a competent facility, management by a multidisciplinary and experienced team, and rehabilitation [10, 11].

The prevalence, management, and outcomes of fatal and nonfatal firearm injuries (F&NFFI) due to the Kivu conflict have not been reported. Therefore, we aimed to quantify the burden of F&NFFIs in the Eastern Democratic Republic of Congo and identify correlates of mortality. The study findings will be used to improve triage and management in our institution and other low-resource settings.

## Methods

This study was authorized by the institutional review board of the Provincial General Reference Hospital of Bukavu. The Provincial General Reference Hospital of Bukavu is a tertiary referral hospital and level 1 trauma center in Bukavu, South Kivu. It offers advanced trauma care thanks to its multidisciplinary team (i.e., at least seven specialty surgeons including two orthopedic-trauma surgeons, two urologic surgeons, two general surgeons, and one neurosurgeon), an equipped and staffed surgical intensive care unit, the implementation of trauma evaluation and management protocols including massive transfusion, on-call trauma surgeon schedule, and Advanced Trauma Life Support. Of note, our institution lacks a cell separator so we always transfuse whole blood. The Congolese Red Cross has designed a “track and transport” system for conflict-related trauma in the Kivu regions. Patients are identified by dedicated “trauma spotters” and transported to our facility by road or air. The system’s biggest flaw is the delay in identifying and tracking trauma patients. As a result, the track-to-transport time significantly increases the delay in reaching specialized trauma care at our facility. Unfortunately, some patients travel in non-medicalized vehicles to get care at our facility.

We included adult F&NFFI patients admitted at our institution between January 01, 2017, and December 31, 2020. We extracted sociodemographic data and clinical [Glasgow coma scale (GCS), heart rate (HR), respiratory rate (RR), systolic blood pressure (SBP), diastolic blood pressure (DBP), temperature (T^o^C), oxygen saturation (SaO_2_), hemoglobin concentration (Hb), shock index (SI), revised Trauma score (RTS), and Kampala Trauma Score (KTS)] data from patient charts.

We computed frequencies and percentages for qualitative variables and measures of central tendency and spread for quantitative variables. Also, we evaluated associations between dependent variables and mortality using the two-sample t-test and Chi-square test of independence. Also, we used multivariable regression analysis to identify confounders among the variables that showed statistical significance during bivariable analyses. A *P*-value < 0.05 was considered statistically significant. Moreover, we illustrated length-of-stay data disaggregated by complication status (i.e., having experienced a complication (or not) during hospitalization) using a time-to-event curve.

## Results

**Fig. 1:**
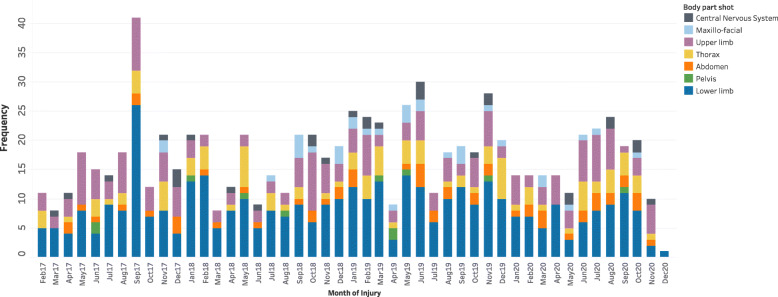
Trend of gunshot injuries and body region injured in the Eastern Democratic Republic of Congo from 2017 to 2020

We admitted 814 adult patients during the study period. There was a gradual annual increase from 2017 (*n* = 183, 22.5%) to 2018 (*n* = 192, 23.6%), and from 2018 to 2019 (*n* = 253, 31.1%). However, the number of injuries dropped to 186 (22.9%) in 2020. The most commonly injured body parts were the lower limbs (*n* = 392, 48.2%), followed by the upper limbs (*n* = 189, 23.2%) (Fig. 1).

The patients were 34.5 (95% CI [33.6, 35.5]) years old on average. They lived 154.4 (95% CI [141.2, 167.5]) km away from the hospital and the mean injury-to-admission time was 4.9 (95% CI [3.6, 6.1]) days. Most patients were male (*n* = 701, 86.1%) and did not have chronic medical conditions (*n* = 805, 98.9%). At admission mean vital sign values were: HR - 90.3 (95% CI [88.9, 91.8]) bpm, RR - 21.8 (95% CI [21.5, 22.1]) cpm, SBP 117.8 - (95% CI [116.3, 119.3]) mmHg, and T^o^C - 36.6 (95% CI [36.6, 36.7]) ^o^C. The mean SI was 0.79 (95% CI [0.78, 0.80]) - 394 patients (48.4%) SI > 0.70.

Most patients had a fracture (*n* = 503, 61.8%) and 501 patients (61.5%) had an open fracture. The mean RTS was 11.8 (95% CI [11.8, 11.9]) and the mean KTS was 14.7 (95% CI [14.6, 14.7]).

**Fig. 2:**
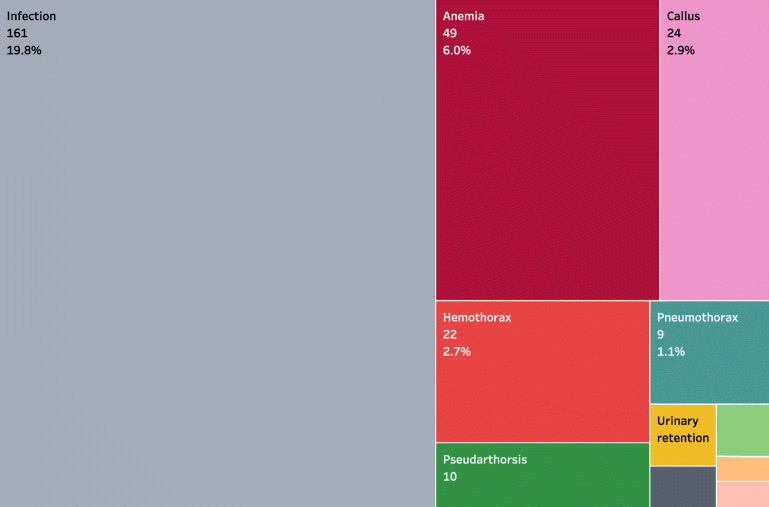
Complications recorded in gunshot injury patients. The first number is the frequency and the second number is the percentage. DVT = Deep vein thrombosis, and Hemoperi = Hemoperitoneum

**Fig. 3:**
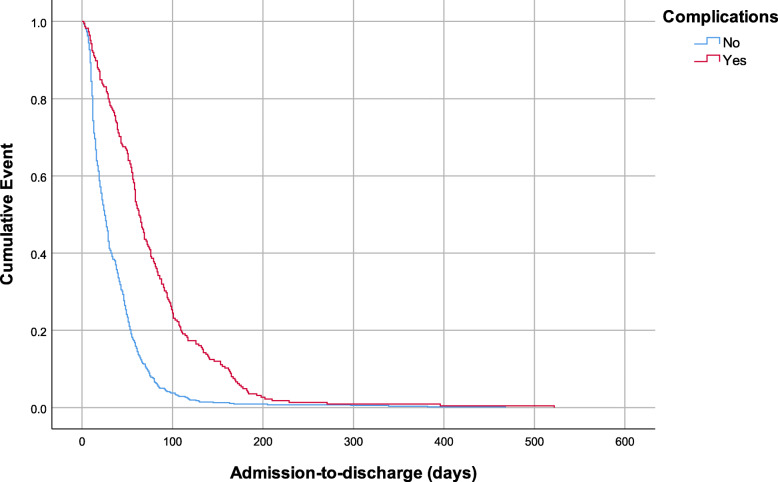
Complications recorded in gunshot injury patients. The first number is the frequency and the second number is the percentage. DVT = Deep vein thrombosis, and Hemoperi = Hemoperitoneum

**Fig. 4:**
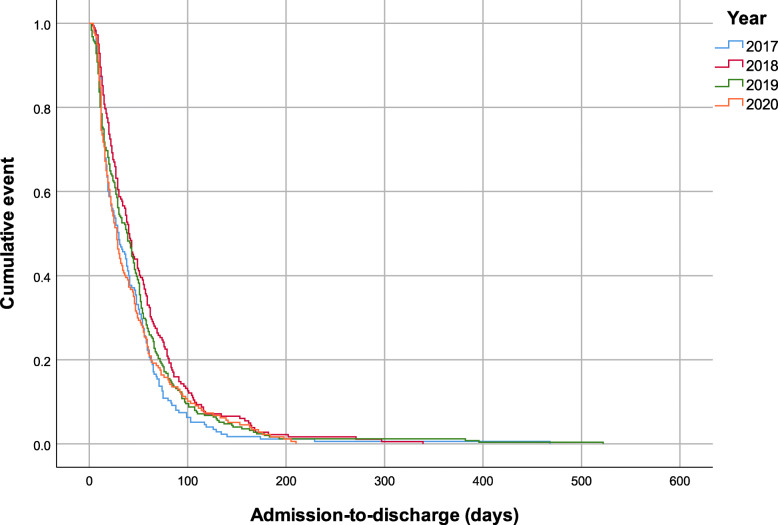
Time-to-event curve showing the length of stay disaggregated by year

All patients received prophylactic antibiotics, the majority (*n* = 785, 96.4%) were treated surgically, and the median length of stay was 34.0 (95% CI [30.4, 37.6]) days. Two hundred and thirty-seven patients (29.1%) presented complications and the most common complications were infections (*n* = 161, 19.8%) and anemia (*n* = 49, 6.0%) (Fig. 2). Of note, patients who had complications had longer median hospital stays than those who did not experience complications (63.0, 95% CI [57.4–68.6] days vs. 25.0, 95% CI [22.4, 27.7] days, *P* < 0.001) (Fig. 3). There were no clinically significant differences in the length of stay over years (Fig. 4).

Twenty-nine patients (3.6%) died during hospitalization. Of these 29, 20 had a SI > 0.70, and patients with fatal injuries had higher SI values than patients with nonfatal injuries (*P* < 0.001). The fatal firearm injury patients had faster HR (*P* = 0.001) and RR (*P* = 0.02). Also, they had lower DBP (*P* = 0.04), SaO_2_ (*P* < 0.001), and hemoglobin values (*P* < 0.001). The differences in RTS and KTS at admission between patients who died and those who did not were minimal and did not show statistical significance (Table 1). Patients who experienced complications were more likely to die; however, there was no evidence that this difference occurred by chance alone (OR = 1.8, 95% CI [0.83, 3.74], *P* = 0.14).

**Table 1:** Determinants of mortality in Congolese firearm injuries patients

Characteristic	Fatal firearm injuries (median, IQR)	Nonfatal firearm injuries (median, IQR)	***P***-value
Age (years)	35.0 (25.0)	32.0 (16.0)	0.06
Distance from the hospital (km)	66.2 (197.5)	82.2 (306.1)	0.24
Injury-to-admission time (days)	1.0 (3.0)	2.0 (3.0)	0.35
Heart rate (bpm)	103.0 (33.0)	88.0 (21.0)	0.001*
Respiratory rate (cpm)	24.0 (8.0)	20.0 (4.0)	0.02*
Systolic blood pressure (mmHg)	116.0 (43.0)	118.0 (20.0)	0.16
Diastolic blood pressure (mmHg)	63.0 (36.0)	72.0 (16.0)	0.04*
Temperature (°C)	36.7 (3.0)	36.5 (1.0)	0.51
SaO_2_ (%)	91.0 (10.0)	98.0 (4.0)	< 0.001**
Hemoglobin concentration (g/dl)	8.9 (2.7)	11.8 (3.0)	< 0.001**
Shock index	0.93 (0.40)	0.74 (0.20)	< 0.001**
Revised Trauma Score	12.0 (0)	12.0 (0)	0.32
Kampala Trauma Score	15.0 (2.0)	15.0 (1.0)	0.46

*P < 0.05, **P < 0.001

After multivariable regression analysis, DBP (β = − 0.39, SE = 0.02, *P* = 0.01), SaO_2_ (β = − 0.15, SE = 0.04, *P* < 0.001), and hemoglobin (β = − 0.30, SE = 0.10, *P* = 0.002) remained statistically significant explanatory variables of mortality (Table 2).

**Table 2:** Multivariable analysis of firearm injury mortality correlates

Correlate	Beta	Standard error	P-value
Constant	15.94	4.69	0.001*
Heart rate (bpm)	0.02	0.01	0.08
Respiratory rate (cpm)	−0.04	0.05	0.42
Diastolic blood pressure (mmHg)	−0.39	0.02	0.01*
SaO_2_ (%)	−0.15	0.04	< 0.001**
Hemoglobin (g/dl)	−0.30	0.10	0.002*

*P < 0.05, **P < 0.001

## Discussion

This is the first study to assess the correlates of F&NFFI mortality among Congolese adults. Most F&NFFI patients at our institution were young adult males with upper or lower limb injuries. They traveled long distances to get specialized care, and about 30% of patients suffered a complication during hospitalization. Complications lengthened hospital stay by more than 5 weeks. Overall, the mortality rate was low but correlated with Hb, DBP, SaO_2_, and SI.

Patients with fatal injuries had lower Hb than patients with nonfatal injuries (8.9 vs. 11.8 g/dl, *P* < 0.001). Anemia and infection were detected at presentation – indicating that they had developed prior to the patients’ presentation at our facility. Low Hb is a reliable indicator of severe bleeding in trauma [12]. Spahn et al. [12] recommend resuscitative measures when Hb falls below 7–9 g/dL. According to these recommendations, most fatally injured patients in our study would have been candidates for erythrocyte transfusion. In contrast, most nonfatal injury patients would not have needed resuscitation because they were hemodynamically stable. Resuscitative measures help prevent hemorrhagic shock, but in the process, they decrease the predictive capacity of Hb by acting as a confounder [13]. In such cases, continuous noninvasive hemoglobin measurement and calculation of the SI are reliable alternatives [14]. Our findings suggest that a Stop the Bleed Educational Initiative in the region may help reduce bleeds significantly.

In low-resource and mass casualty settings, strategic allocation of medical resources is critical to maximizing survival. To date, multiple triage scoring protocols have been utilized for injury descriptions, outcomes, and mortality prediction including the RTS, a physiologic scoring system; the KTS, an anatomic and physiologic scoring system; and the Injury Severity Score (ISS), an anatomic scoring system [15]. The RTS and ISS have better predictive values in high-resource settings [15]. This is because they require more complex calculations and diagnostic tools, which may not be available in low-resource settings [16]. The KTS was developed to overcome this barrier [17]. Neither the RTS nor the KTS was correlated with patient outcomes in this study. The RTS and KTS use similar variables (ex: neurologic status, SBP, and RR) to evaluate trauma severity; however, none of these scoring systems has been specifically described as the standard scale [16, 18]. This is supported by the lack of consistent research results regarding the preferred system in different resource settings. Thus, an adequate scoring system in a particular setting does not just entail collating systems that have been successful in similar settings but using one that is easy to integrate and fits with the available resources [16, 19].

Unlike the RTS and KTS, the SI was correlated with mortality. The SI is the HR divided by the SBP. An SI > 0.70 suggests acute hypovolemia, and higher values are associated with a worse prognosis [20]. Of note, the majority of fatal firearm injury patients had an SI > 0.70. The statistically significant association between mortality, SI, DBP, SaO_2_, and Hb suggests the primary cause of mortality in our patient cohort was tissue hypoperfusion following circulatory failure. Of note, the causes of death in our cohort were septic shock and hypovolemic shock. In the context of circulatory failure, the SI is difficult to interpret because the HR was normocardic and SBP was > 100 mmHg. Having tested the RTS and KTS with little success, we have opted to calculate and evaluate other trauma scores in our institution. We hope to find one that makes sense clinically in this setting. If we do not find one, we intend to develop a score based on historical data and to then test it prospectively for validation.

Local clinical management systems should assess hemorrhagic shock, bleeding control, and patient outcomes, especially in the prehospital setting. The first 10 min (“platinum ten minutes”) following a firearm injury are critical for managing life-threatening bleeding [12, 21]. The most prevalent injuries in this study were firearm injuries to the extremities. Traumatic hemorrhage of the extremities can be controlled at the site of injury with tourniquets [12]. Our patients lived more than 154 km away from our facility and were admitted almost 5 days after the injury. Fatally injured patients lived closer to our facility and had shorter injury-to-admission times. This suggests that more severely injured patients living far away from our institution were less likely to arrive at our facility. Immediate transfer of severely injured patients to competent trauma facilities reduces morbidity and mortality [12]. Unfortunately, our region has no formal referral pattern, and our institution is the only level 1 trauma center in the South Kivu region. South Kivu has a surface area of 65,070 km^2^ (about the same size as West Virginia) and more than 5.8 million inhabitants [22]. As a result, most F&NFFI patients seek care in under-resourced facilities and are transferred to non-ambulance vehicles. Also, the ongoing conflicts have created insecurity deterring patients from seeking care. Health system strengthening efforts to build referral systems and capacity can help reduce delays and barriers to getting timely and safe trauma care [12]. For example, establishing a hub-and-spoke referral model in the South Kivu province and Advanced Trauma Life Support training of healthcare workers in trauma hubs.

The complication rate was 29.1%, and infection was the most common complication. The infection rate was low considering how late the patients presented at our facility. There is limited pre-hospital data but we surmise that most patients received large spectrum antibiotics at district hospitals before they presented at our center. This hypothesis is supported by the fact that most Congolese district hospitals do not have the resources to repair open fractures but have access to large spectrum antibiotics. Antibiotic prophylaxis and debridement reduce the infection rate in F&NFFI independently [25, 26]. Furthermore, antibiotic prophylaxis is more effective when combined with surgical treatment; however, the duration of administration does not appear to reduce infection rates [27]. In this study, complications significantly increased the length of stay and mortality rate. These findings further highlight the necessity for aseptic manipulation of the injured patients when possible and the strict implementation of antiseptic measures.

As discussed above, delays to safe care cause excess morbidity and mortality. In 1994 Thadeus and Maine [23] described the three-delay model. The model was originally described for access to maternal care but has since been expanded to other forms of care, including emergency and trauma surgery [24]. In this study, we assessed the impact of two of the three delays to care (i.e., i. The second delay - delay in reaching care, and ii. The third delay - delay in getting care). We lacked data to assess the magnitude and impact of the first delay (i.e., delay in seeking care). We believe it is essential that future studies evaluate the first delay especially considering the impact of the third and second delays on patient outcomes. The second and third delays have been assessed extensively in previous studies, while the first delay remains understudied [24]. The first delay can be studied using household surveys, qualitative analyses, patient interviews, and medical record reviews [24]. These studies will give an insight into patient beliefs, attitudes, and practices towards F&NFFIs and their management. The findings of these studies will inform health system strengthening strategies in Eastern DRC and help increase access to timely care, thereby reducing the burden of F&NFFIs.

The burden of F&NFFI in Eastern Congo is enormous and has been increasing each year except during 2020. It is possible that the decrease in 2020 was the result of stay-at-home orders. The number of cases was stable in the first months of 2020 when the first COVID-19 case in Congo was identified, and the government issued stay-at-home orders [28]. However, there was a sharp rise from June to August 2020. This rise might be associated with increasing weariness and mounting frustration as the stay-at-home orders were extended. New Zealand and Italy have noted decreasing F&NFFIs during the pandemic [29, 30]. However, there have been substantial increases in firearm purchases and F&NFFI in the United States [31]. This increase has been attributed to prolonging stay-at-home orders and their negative impact on mental health and finances [31].

Local and global surgeons can help decrease the injury burden of the Kivu conflict through research, advocacy, education, and trauma service delivery. More research is needed to understand patient referral pathways and trauma care processes in need of quality improvement. Next, the research findings should be disseminated locally and internationally to the public, policy makers, and belligerent parties involved in the conflict. These communications should include holistic health systems action-plans with clear targets and timelines for monitoring and evaluation. Furthermore, trauma care stakeholders should design and implement capacity-building programs for laypeople and district hospitals.

### Limitations

The data used in this study were from a single-center and its findings should be interpreted with caution. For example, we cannot ascertain that Congolese patients are mainly shot in lower extremities because our data is hospital-based and devoid of pre-hospital data. However, we note that other authors have found similar distributions of injury. The general understanding of this phenomenon is that patients who are shot in the lower extremities have more favorable vital outcomes and are more likely to survive a delayed transfer to a referral hospital. Hence, it is probable that we missed out on the severe F&NFFI cases especially those remote from our facility. Next, the mortality data presented were unstandardized in-hospital data. As such, we lacked follow-up data to assess long-term mortality. Notwithstanding, we reported 30-day data for most patients because the 95% CI lower limit of the length of stay was 30.4 days.

## Conclusion

The Kivu armed conflict in Eastern Congo is causing avertable morbidity and mortality. The burden of the conflict is on the rise, and young adult males are disproportionately affected. We assessed the association between mortality in adult F&NFFI patients and routine clinical measurements, the SI, RTS, and KTS. Mortality is associated with Hb, DBP, and SaO_2_. Also, the SI is a better explanatory variable than the RTS or KTS. Health systems strengthening is needed to reduce delays in reaching and getting care in Eastern Congo. These improvements should focus primarily on prehospital care. More importantly, stakeholders must meet to find a peaceful solution to the conflict. A peaceful solution should help reduce the burden of F&NFFI in Congo.

## Data Availability

The datasets used and/or analyzed during the current study are available from the corresponding author on reasonable request.

## Abbreviations

CI: Confidence interval
DBP: Diastolic blood pressure
DRC: Democratic Republic of Congo
F&NFFI: Fatal and nonfatal firearm injury
GCS: Glasgow coma scale
Hb: Hemoglobin concentration
HR: Heart rate
KTS: Kampala trauma score
OR: Odds ratio
RR: Respiratory rate
RTS: Revised trauma score
SaO_2_: Oxygen saturation
SBP: Systolic blood pressure
SI: Shock index
T^o^C: Body temperature
